# Exploring profile and potential influencers of vaginal microbiome among asymptomatic pregnant Chinese women

**DOI:** 10.7717/peerj.8172

**Published:** 2019-12-10

**Authors:** Yining He, Yun Huang, Zhengyin Zhang, Fengping Yu, Yingjie Zheng

**Affiliations:** 1 Department of Epidemiology, School of Public Health, Fudan University, Shanghai, China; 2 Department of Clinical Laboratory, Shanghai Punan Hospital of Pudong New District, Shanghai, China; 3 Department of Obstetrics and Gynecology, Shanghai Punan Hospital of Pudong New District, Shanghai, China; 4 Key Laboratory of Health Technology Assessment, National Health Commission (Fudan University), Shanghai, China; 5 Key Laboratory of Public Health Safety, Ministry of Education, School of Public Health, Fudan University, Shanghai, China

**Keywords:** Vaginal microbiome, Pregnant women, Body mass index, Passive smoking, Vaginal cleanliness

## Abstract

**Background:**

This study was designed to explore the profile and potential influencers of the vaginal microbiome (VMB) among asymptomatic pregnant Chinese women and its possible association with pregnancy outcomes.

**Methods:**

A prospective study was conducted among pregnant Chinese women receiving regular prenatal care at a hospital in Shanghai, China from March 2017 to March 2018. Vaginal swabs were obtained from 113 asymptomatic pregnant women in mid-pregnancy and sequenced by the V3–V4 region of 16S rRNA on an Ion S5™ XL platform. Demographic characteristics and major pregnancy outcomes were collected through questionnaires and electronic medical records.

**Results:**

The predominant vaginal community state types (CSTs) were CST I (45.1%) and CST III (31.9%). Participants were divided into a lactobacilli-dominant group (LD, CST I/II/III/I–III/V, *n* = 100, 88.5%) and a less lactobacilli-dominant group (LLD, CST IV-A/B, *n* = 13, 11.5%). Women in the LLD group showed an increased alpha diversity [median (interquartile range, IQR): 2.41 (1.67, 2.49) vs. 0.30 (0.17, 0.59), *P* < 0.001], which was related to a lower pre-pregnancy body mass index (BMI) (*P* = 0.012), and a greater instance of passive smoking (*P* = 0.033). The relative abundance of *Lactobacillus* was correlated positively with the pre-pregnancy BMI (*r* = 0.177, *P* = 0.041), but negatively with passive smoking (*r* =  − 0.204, *P* = 0.030).

**Conclusion:**

The vaginal flora of asymptomatic pregnant Chinese women was mostly dominated by *Lactobacillus crispatus* and *L. iners*. A lower BMI and greater instance of passive smoking may contribute to a less lactobacilli-dominant VMB. However, a larger sample size is needed.

## Introduction

The vaginal microbiome (VMB) is known to play an essential role in women’s reproductive health. Previous culture-based methods recognized the genus *Lactobacillus* to be the dominant bacterium in the vagina (Rogosa & Sharpe, 1960). These bacteria can maintain the balance of the vaginal microenvironment through the production of lactic acid from vaginal glycogen to keep a low pH value (usually < 4.5). They are also involved in the production of hydrogen peroxide (H_2_O_2_) and acidolin, which prevents the overgrowth of other intrinsic bacterial and the invasion of foreign pathogens (Aroutcheva et al., 2001; Miller et al., 2016; Mitchell et al., 2013).

The recently developing molecular technologies have enabled researchers to get a better understanding of the VMB (Fettweis et al., 2012; Mendz, Kaakoush & Quinlivan, 2016). With the help of the next generation of sequencing (NGS), Ravel et al. (2011) was able to cluster the VMB of asymptomatic child-bearing women into five vaginal community state types (CSTs). CST I, II, III, and V were dominated by *L. crispatus*, *L. gasseri*, *L. iners* and *L. jensenii* (with an abundance usually >90%) respectively, while CST IV represented a more diverse profile with a lesser abundance of *Lactobacillus* and could be further divided into two subtypes, CST IV-A (a coexistence of *Lactobacillus* and some anaerobes) and CST IV-B (mostly dominated by *Gardnerella*, *Atopobium*, *Prevotella*, and others) (Brotman et al., 2014b; Huang et al., 2015; Romero et al., 2014). Though the dominant bacteria in CST IV could also produce lactic acid, the pH value is slightly higher (Gajer et al., 2012; Ravel et al., 2011). Moreover, the VMB needs to maintain a lower diversity to obtain the microbial balance in the vagina, which is unlike other sites throughout the body, such as the gut and oral cavity (Huang et al., 2014).

The composition and abundance of the VMB could be affected by various factors, such as intrinsic genotypes, host behaviors, and health conditions (Greenbaum et al., 2019; Lewis, Bernstein & Aral, 2017; Zhang et al., 2018). Ethnicity may be the most influential of all of these factors. In Ravel’s study, White women were mostly dominated by CST I (45.4%), Asians by CST III (42.7%), and Blacks and Hispanics by CST IV (40.4% and 38.1%) (Ravel et al., 2011). Fettweis et al. (2014) further confirmed the lesser abundance of *Lactobacillus* in African Americans. In addition, the pH value of African descendants was higher than that of Whites and these women were more likely to develop bacterial vaginosis (BV).

Compared with non-pregnant women, the VMB of pregnant women exhibits a lower diversity and abundance of flora but a more stable community (Aagaard et al., 2012; Husain et al., 2014; Walther-Antonio et al., 2014). Since the maternal VMB during pregnancy could largely affect the next generation, researchers are interested in discovering how the VMB could affect pregnancy outcomes. The rich progress in the research of the VMB during pregnancy has been driven by the Human Microbiome Projects in recent years (Fettweis et al., 2019; Serrano et al., 2019). Researchers have found that the lesser abundance of *Lactobacillus* in the VMB was more likely to lead to the development of adverse pregnancy outcomes, such as preterm birth, still birth, and late miscarriage (Baqui et al., 2019; DiGiulio et al., 2015; Fettweis et al., 2019; Hyman et al., 2014; Tabatabaei et al., 2019). Results have also shown that race or ethnicity may modify the association of the VMB and preterm birth mostly among women of African and European ancestry (Callahan et al., 2017; Wheeler et al., 2018). However, the association among Asian populations remains unclear.

Studies involving local Asian women have been implemented in Japan, Korea, Thailand, and China (Chen et al., 2017; Hong et al., 2016; Matsumoto et al., 2018; Sirichoat et al., 2018), but few have well investigated the VMB of pregnant women using NGS (Kim et al., 2017; Matsumoto et al., 2018), especially among Chinese populations. Thus, in order to gain a more comprehensive understanding to VMB and to see if there was an association of VMB with pregnancy outcomes, we undertook this study to probe into the profile of the VMB among asymptomatic pregnant Chinese women.

## Materials & Methods

### Participants and procedures

A prospective study was conducted among pregnant Chinese women receiving regular prenatal care at Shanghai Punan Hospital of Pudong New District in Shanghai, China from March 2017 to March 2018. The study was reviewed and approved (IRB#2017-01-0608) by the ethical committee of the School of Public Health, Fudan University (IRB00002408 & FWA00002399).

Participants were recruited at their first prenatal visit. The inclusion criteria included: (a) aged 16 years or older with a singleton pregnancy, (b) gestational week earlier than 28 weeks, and (c) ability to complete the study procedure. The exclusion criteria included: (a) the use of antibiotics within four weeks prior to recruitment, (b) sexual intercourse 48 h prior to sampling, and (c) having a severe illness (such as liver or heart disease). After informed consents, participants were asked to complete a structured questionnaire on their sociodemographic characteristics, medical and reproductive history, and lifestyle data, and were then followed up for their major pregnancy outcomes including delivery (gestational weeks of delivery, delivery mode) and infant information (gender, birth weight) through hospital electronical records.

### Sample collection and clinical examination

Vaginal samples were taken by skilled obstetricians from the posterior fornix using sterile swabs. Two swabs were obtained from each participant. One was placed in a tube without any buffer and stored at −80 °C before use and the other was used for clinical inspection with a wet mount. The results were reported by an experienced laboratory staff (Huang). The laboratory examination included the presence of vaginal inflammation and standardized I-IV grades of “vaginal cleanliness” were used by wet mount microscopy (Shang, Wang & Shen, 2014), which was a composite indicator based on grading bacillus, coccus, epithelial cells, and leukocytes per high power (HP). Higher grades imply a presence of vaginal inflammation (Table S1).

### DNA extraction, 16S rRNA amplification and sequencing

The total genome DNA from samples was extracted using a mixture of cetyltrimethylammonium bromide (CTAB) and sodium dodecyl sulfate (SDS) methods. The hypervariable V3–V4 region of the 16S rRNA gene (Primer: 341F-CTAYGGGRBGCASCAG; 806R-GGACTACNNGGGTATCTAAT) was amplified with the sample-specific barcodes and then sequenced on an Ion S5™ XL platform (Novogene Co., Ltd., Beijing, China), generating results of 600 bp single-end reads.

### Bioinformatic and statistical analyses

The upstream analysis of the sequencing data was conducted on the QIIME 2 software platform (Quantitative Insights into Microbial Ecology 2, Version 2018.11, http://qiime2.org) with its plugins *DADA2*, *feature-table*, *feature-classifier* and *phylogeny*. Single-end reads were assigned to samples based on their unique barcodes and truncated by cutting off the barcodes and primer sequences. Quality controls were performed and sequences were denoised with the *DADA2*. After filtering the low abundance of features (reads <10) and omitting chimeras, the representative sequences for amplicon sequence variants (ASVs) were screened for annotation using the SILVA-based training classifier database. The majority of the *Lactobacillus* genus could not reach the species level because of the short read lengths obtained by high-throughput sequencing, but it was essential for further analysis in studies of VMB (Van Der Pol et al., 2019).Therefore the BLAST (Basic Local Alignment Search Tool) database at the National Center for Biotechnology Information website (http://blast.ncbi.nlm.nih.gov) was utilized with a minimum support threshold of 80%, and the top hit was selected as the species level. If multiple top hits with exactly the same values are found, we expressed them as “*Lactobacillus sp.*” and numbered them in order.

After the ASV table was obtained, further analyses were performed using R 3.5.2 software for Windows (R Core Team, 2018) and the packages vegan, phyloseq, ggplot2, and pheatmap. Alpha diversity showing the richness and evenness within the subjects was computed using the Shannon index (SID). We also calculated the Bray–Curtis distance as beta diversity for clustering CSTs according to DiGiulio et al. (2015): extracting the most significant Principal Coordinates Analysis (PCoA) eigenvectors to form the distance matrix, applying the partitioning around medoids algorithm, and determining the number of clusters form the gap statistic (*k* = 7). A heatmap was displayed for visualizing. The linear discriminate analysis coupled with effect size measurements (LEfSe) was performed to identify differential species among different characteristic groups of participants with a linear discriminant analysis (LDA) score >2.0 considered as significant.

Continuous variables were presented using mean  ± standard deviation (SD) or the median [interquartile range (IQR)], while discrete variables were presented using numbers and proportions. Referring to previous studies, the subjects were divided into two distinct subgroups according to the vaginal dominance of *Lactobacillus*. For univariate analyses of participant characteristics, alpha diversity, and relative abundance of taxa between groups, one-way ANOVA, the Mann–Whitney *U* test and Pearson *χ*^2^ test (or Fisher’s exact test) were used to compare the means, medians, and proportions, respectively. The relationships of two continuous variables, including the relative abundance of taxa, alpha diversity, pre-pregnancy body mass index (BMI), passive smoking (days per week), and gestational ages in weeks were investigated with Spearman’s *rho*. The level of a significant difference was set at a two-sided 0.05.

## Results

### Participant characteristics

**Figure 1:**
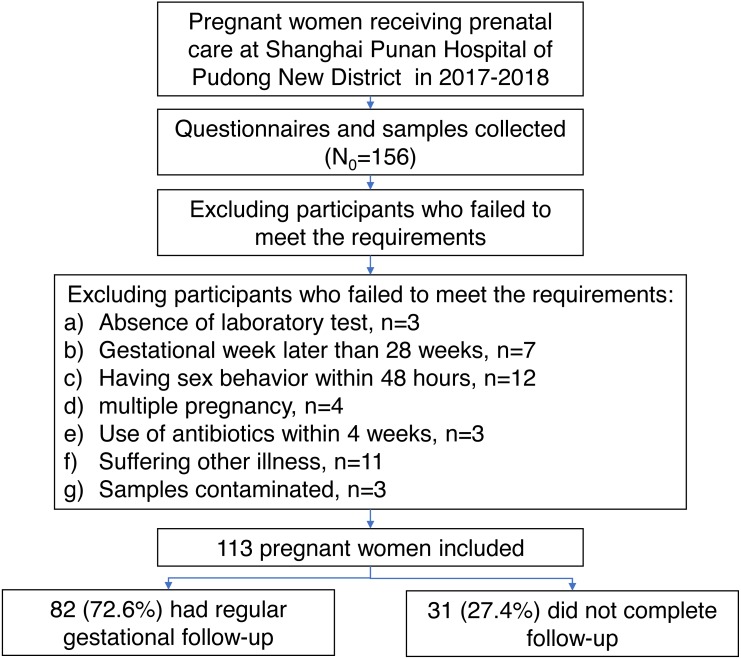
The flowchart shows the selection of participants enrolled in this study with consent informed.

During the period from March 2017 to March 2018, a total of 156 women were recruited at their first prenatal visit in the Shanghai Punan Hospital of Pudong New District in Shanghai, China. After a careful screening, 113 pregnant women met the inclusion/exclusion criteria (Fig. 1). All subjects were Han Chinese in mid-pregnancy, with ages ranging from 17 to 34 years old (mean ± SD: 25.69 ± 3.69) and with gestational ages of 12.42 to 26.71 weeks (mean ± SD: 16.68 ± 2.76). After a regular gestational follow-up, the delivery data of 82 women (72.56%) was collected at Punan Hospital. The gestational age at birth ranged from 35.86 to 41.43 weeks (mean ± SD: 39.40 ± 1.23). The demographic characteristics of women with delivery data collected and not collected were comparable, except for the proportion of vaginal inflammation (Table S2).

### Vaginal microbiome profiles

A total of 113 vaginal samples were sequenced, generating 3,671,402 raw reads. Chimeric and low abundance of features (reads < 10) were filtered out, leaving 3,644,890 reads with an average of 32,255 reads per sample (ranging from 1,424 to 52,991 reads). Among annotated taxa, *L. crispatus* generated the most reads at 2,279,244, followed by *L. iners* and *L. gasseri*. *Megasphaera*, *Dialister* and *Aerococcus* were the top three genera following the *Lactobacillus* genus.

### CSTs definition

**Figure 2:**
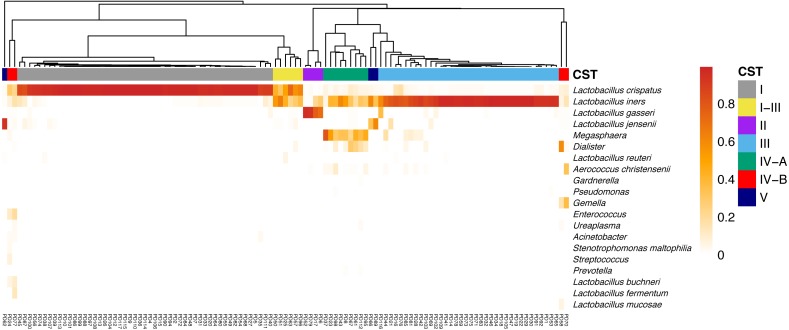
The heatmap indicates the relative abundance of top taxa in all samples. The CSTs were clustering using Bray–Curtis indices, and was annotated on the top of the heatmap. Notes. CST, community state types.

Seven CSTs were identified using the clustering Bray–Curtis distance matrix. Five of them were dominated by *Lactobacillus.* Of these, *L. crispatus* dominated CST I had the highest proportion (51/113, 45.1%), followed by *L. iners,* which dominated CST III (36/113, 31.9%). *L. gasseri* and *L. jensenii* dominated CST II and V took up only 3.5% (4/113) and 2.7% (3/113) of the subjects, respectively. A I-III type (6/113, 5.3%) contained almost equal abundance of *L. crispatus* and *L. iners*. The other two CSTs were divided into CST IV-A (coexistence of *L. iners* and *Megasphaera*, *Dialister*, etc., 9/113, 8.0%) and IV-B (diversity, 4/113, 3.5%) according to their lower abundance of *Lactobacillus*. The relative abundance of the top taxa in different CSTs was displayed in a heatmap for visualizing (Fig. 2).

### LD and LLD groups

**Figure 3:**
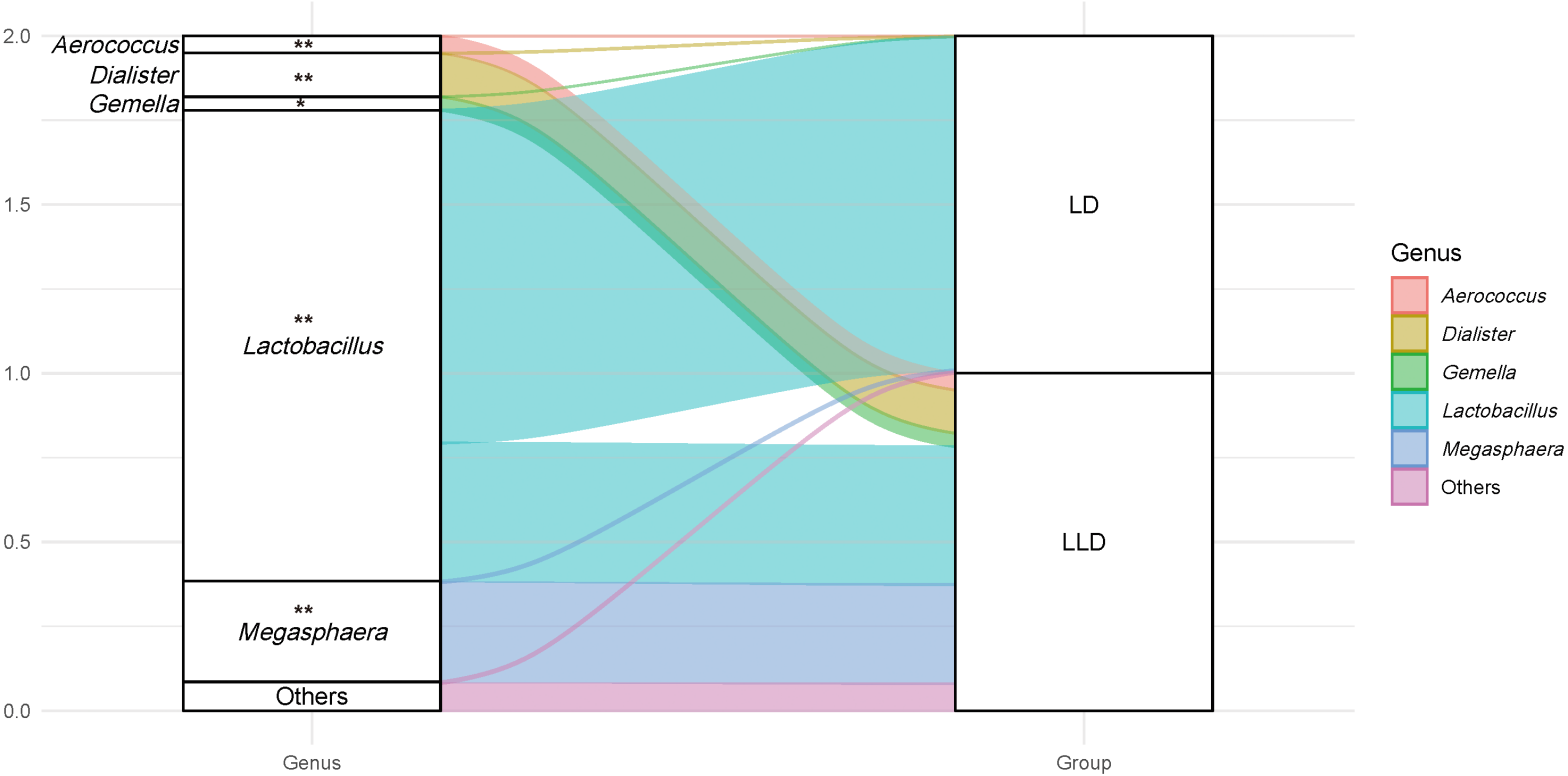
An alluvial diagram showing the relative abundance of top taxa in LD and LLD groups. The genus of *Megasphaera*, *Dialister*, *Aerococcus*, *Gardnerella*, and *Gemella* were detected more frequently in the LLD group (*P* < 0.05 for all). Genus with abundance less than 1% is not shown (*Gardnerella*). Notes. * indicates *P* value < 0.05, and ** indicates *P* value < 0.001. LD, lactobacilli-dominant group; LLD, less lactobacilli-dominant group.

The relative abundance of the *Lactobacillus* genus in CST I/II/III/I–III/V were all higher than 90%, thus the participants were divided into two groups: The lactobacilli-dominant group (LD, CST I/II/III/I–III/V, *n* = 100, 88.5%), and the less lactobacilli-dominant group (LLD, CST IV-A/B, *n* = 13, 11.5%). More detailed bacterial abundance data from different CSTs are shown in Table S3. The relative abundance of other genera was also compared between groups. The genus of *Megasphaera*, *Dialister*, *Aerococcus*, *Gardnerella*, and *Gemella* were detected more frequently in the LLD group (*P* < 0.05 for all, Fig. 3). The sociodemographic, medical and reproductive history, lifestyle characteristics, and major pregnancy outcomes of subjects were compared between the two groups (Table 1).

Results showed that women in the LLD group had a lower maternal pre-pregnancy BMI (especially the proportion of BMI lower than 18.5 kg/m^2^) (mean ± SD: 19.16 ± 2.60 vs. 21.16 ± 2.68, *P* = 0.012; 38.5% vs. 13.0%, *P* = 0.045) and participated in more passive smoking (>3 days per week) (38.5% vs. 15.0%, *P* = 0.033). There were no differences in the pregnancy outcomes between the groups (*P* > 0.05 for all). Nevertheless, women in the LLD group tended to have a higher proportion of vaginal inflammation (but not higher than CST I–III and III, Table S4), active smoking, and preterm birth in contrast with those in the LD group.

### Comparative analysis of alpha diversity

A significantly increased SID was observed in the LLD group (median (IQR): 2.30 (1.69, 2.48) vs. 0.28 (0.17, 0.57), *P* < 0.001). To investigate the potential influential factors of alpha diversity within the subjects, SID was further compared among selected characteristics (Table 1). Results revealed that the presence of vaginal inflammation was correlated with a higher SID when compared with that of lower grades (median (IQR): 0.55 [0.29, 1.28] vs. 0.29 [0.18, 0.82], *P* = 0.017). Women with a lower BMI (*P* = 0.181) and who participated in more passive smoking (*P* = 0.262) also had a tendency toward higher diversity, in accordance with the result of CST distribution, although the difference did not achieve statistical significance at 0.05. With the growing gestational age of weeks at enrollment, the alpha diversity showed a declining trend (*P* = 0.120).

**Table 1:** LD/LLD Groups and SID among relevant characteristics of subjects.

Characteristics (*N* = 113)	LD Group (*n* = 100)	LLD Group (*n* = 13)	*P*	SID	*P*
	CST I/II/III/I–III/V	CST IV-A/B			
**Sociodemographic**					
Age (mean ± SD) (years old)	25.70 ± 3.78	25.62 ± 3.07	0.938	–	–
<18	1(1.0)	0(0.0)	>0.99^*^	0.30 [0.30, 0.30]	0.984
18–25	36(36.0)	5(38.5)		0.32 [0.21, 0.90]	
25–35	63(63.0)	8(61.5)		0.32 [0.18, 0.89]	
Gestational age of weeks at enrollment (mean ± SD) (weeks)	16.78 ± 2.86	15.92 ± 1.64	0.294	–	–
12–18	79(79.0)	12(92.3)	0.810^*^	0.33 [0.21, 0.97]	0.120
18–24	17(17.0)	1(7.7)		0.29 [0.17, 0.90]	
24–28	4(4.0)	0(0.0)		0.17 [0.13, 0.21]	
Education status			0.737^*^		0.622
Middle school and lower	25(25.0)	4(30.8)		0.31 [0.21, 0.57]	
High school and higher	75(75.0)	9(69.2)		0.32 [0.19, 0.95]	
Economic status (RMB per year)			0.459		0.777
<100,000	43(43.0)	7(53.8)		0.32 [0.21, 0.83]	
≥100,000	57(57.0)	6(46.2)		0.30 [0.18, 1.01]	
**Medical and reproductive history**					
Maternal pre-pregnancy BMI (mean ± SD) (kg/m^2^)	21.16 ± 2.68	19.16 ± 2.60	0.012	–	–
Underweight (<18.5)	13(13.0)	5(38.5)	0.045^*^	0.49 [0.27, 1.24]	0.181
Normal weight (18.5–24.0)	71(71.0)	7(53.8)		0.28 [0.18, 0.80]	
Overweight and obese (>24.0)	16(16.0)	1(7.7)		0.48 [0.21,0.84]	
Unipara	69(69.0)	8(61.5)	0.752^*^	0.32 [0.21, 0.88]	0.777
No	31(31.0)	5(38.5)		0.30 [0.16, 1.02]	
Presence of inflammation (Grade III/IV)	22(22.0)	4(30.8)	0.492^*^	0.55 [0.29, 1.28]	0.017
No	78(78.0)	9(69.2)		0.29 [0.18, 0.82]	
Previous adverse pregnancy outcomes	24(24.0)	3(23.1)	>0.99^*^	0.25 [0.15, 1.06]	0.313
No	76(76.0)	10(76.9)		0.33 [0.21, 0.87]	
**Lifestyle**					
Vaginal douching	34(34.0)	3(23.1)	0.541^*^	0.27 [0.20, 0.70]	0.407
Never	66(66.0)	10(76.9)		0.35 [0.20, 1.01]	
Active smoking	6(6.0)	2(15.4)	0.230^*^	0.33 [0.22, 1.15]	0.538
Never	94(94.0)	11(84.6)		0.31 [0.19, 0.88]	
Passive smoking (>3 days per week)	15(15.0)	5(38.5)	0.033^*^	0.58 [0.21, 1.31]	0.262
≤3 days per week	85(85.0)	8(61.5)		0.30 [0.19, 0.80]	
Drinking	8(8.0)	1(7.7)	>0.99^*^	0.21 [0.16, 0.33]	0.109
Never	92(92.0)	12(92.3)		0.32 [0.21, 0.95]	
**Alpha diversity**					
Shannon index (median [IQR])	0.28 [0.17, 0.57]	2.30 [1.69, 2.48]	<0.001	–	–
**Pregnancy outcomes (*N*′ = 82)**	**72(72.0)**	**10(76.9)**	>0.99*	**–**	–
Delivery mode			0.742^*^		0.258
Cesarean	31(43.1)	5(50.0)		0.28 [0.17, 0.66]	
Vaginal delivery	41(56.9)	5(50.0)		0.37 [0.19, 1.00]	
Gestational age of weeks at birth			0.327^*^		0.212
Preterm birth (<37 weeks)	2(2.8)	1(10.0)		0.88 (0.59,1.57)	
Term birth (≥37 weeks)	70(97.2)	9(90.0)		0.31 [0.17, 0.89]	
Fetus gender			0.735^*^		0.512
Male fetus	29(40.8)	5(50.0)		0.36 [0.20, 0.96]	
Female fetus	42(59.2)	5(50.0)		0.30 [0.18, 0.89]	
Birth weight			>0.99^*^		0.445
Low birth weight	4(5.6)	0(0.0)		0.52 [0.17, 0.89]	
Normal birth weight	62(87.3)	100(100.0)		0.32 [0.21, 1.04]	
Large for birth weight	5(7.0)	0(0.0)		0.28 [0.13, 0.46]	

**Notes.**

*P*-values were calculated using chi-squared or Fisher’s exact analysis (*) for assessment of association of frequency between groups and the Mann-Whitney *U*-Test for comparison of means and medians.

LD lactobacilli-dominant group LLD less lactobacilli-dominant group SID Shannon index CST community state type SD standard deviation BMI Body Mass Index IQR interquartile range

### Bioinformatic trends analysis

To further explore the bioinformatic trends of alpha diversity (SID) and relative abundance of taxa with age, maternal pre-pregnancy BMI, passive smoking (days per week), and gestational age of weeks at enrollment, correlation calculations using Spearman’s *rho* were carried out. Results showed that the maternal pre-pregnancy BMI and the relative abundance of *Lactobacillus* correlated positively (*r* = 0.177, *P* = 0.041) at genus level, but not with any individual species. Moreover, as the days of passive smoking per weeks increased, the abundance of *Aerococcus* also increased (*r* = 0.309, *P* = 0.001), but *Lactobacillus* decreased (*r* =  − 0.204, *P* = 0.030). No statistically significant correlations existed between other pairwise variables.

### Differential abundance of taxa

LEfSe results distinguished the differential taxa among selected characteristics. The VMB of women with vaginal inflammation revealed more *L. iners*, *Dialister*, *Enterococcus*, and *Aerococcus*, and less *L. crispatus*. Smoking also affect the composition of VMB: active smokers have a higher abundance of *Megasphaera* and *Prevotella*, while passive smokers have more *Aerococcus*. BMI groups and selected pregnancy outcomes did not reveal any specific bacteria species.

## Discussion

Overall, this prospective study revealed the predominance of CST I and III by investigating the profile of VMB among 113 asymptomatic pregnant Chinese women. Lower pre-pregnancy BMI and more passive smoking were correlated with a less lactobacilli-dominant VMB. No significant findings were associated with pregnancy outcomes.

Earlier studies have reported that the distribution of CSTs was largely affected by race, and the VMB of Asian women was generally dominated by CST III (Ravel et al., 2011; Zhou et al., 2010). Our study found that CST I was predominant among pregnant Chinese women, followed by CST III. This result, although slightly different, was in line with studies carried out among Korean and Japanese pregnant women (Kim et al., 2017; Matsumoto et al., 2018), implying the possibility of a diverse profile of VMB among Asian women due to genetic variations or other environmental factors such as socioeconomic status. We also identified a I–III type containing an equal abundance of *L. crispatus* and *L. iners* among this population, which was rarely reported in previous studies. Gajer et al. (2012) once divided this type into CST IV-A, but both the composition and the diversity (both alpha and beta) of CST I–III and IV-A were dissimilar. Perhaps the definition of CSTs should be on the basis of dissimilitude.

Apart from race, the composition of the VMB could be affected by other factors (Fettweis et al., 2014; Jasarevic et al., 2017). In Wen’s study, BMI was significantly correlated to the structure of the microbial community and negatively correlated with the presence of *Mycoplasma* and BV-associated bacterium-2 (BVAB2) (Wen et al., 2014). Our study noted a similar finding that women with a lower BMI (particularly <18.5 kg/m^2^) tended to harbor CST IV-A and IV-B, which are the less lactobacilli-dominant type of VMB. Moreover, a positive correlation was observed between BMI and the genus of *Lactobacillus*. This result was in accordance with the study by Mirmonsef et al. (2014), that BMI was positively associated with vaginal glycogen and a higher abundance of lactobacilli. Lower BMI may have lower concentrations of vaginal glycogen, leading to the lack of lactobacilli*.*

Smoking is also an important affecting factor. Women who were active smokers were reported as being prone to having higher proportions of BV-associated bacteria (Brotman et al., 2014a; Fettweis et al., 2014; Ryckman et al., 2009). A recent study by Nelson et al. (2018) reported an altered vaginal tract metabolomic profile among smokers, with higher agmatine, cadaverine, putrescine, tryptamine and tyramine, which were known to affect the virulence of infective pathogens and contribute to vaginal malodor. Notably, our study observed a different VMB composition among women who practiced more passive smoking (>3 days per week) instead of active smoking. Moreover, with the days of passive smoking per weeks increased, the abundance of *Aerococcus* (a BV-associated bacterium) also increased and *Lactobacillus* decreased. However, the differential taxa detected by LEfSe were not the same with women who actively smoked, leading to the issue of whether active and passive smoking had a similar mechanism that affected VMB. To achieve favorable pregnancy outcomes, pregnant women with either active or passive smoking exposures should remain under close scrutiny.

Although our study did not perform the clinical examination for bacterial vaginosis (BV) and aerobe vaginitis (AV), we used the index of “vaginal cleanliness” grades, which is used routinely as an indicator in Chinese hospitals to suggest the potential inflammation of the vagina. It is used as a pilot screening for BV and AV because of its low cost. Researchers have investigated its possible implications for BV and found that higher grades exhibited higher risks of BV (Lin, Wei & He, 2008). LEfSe results showed the relevance of higher grades with a greater abundance of *Aerococcus, Enterococcus*, and *Dialister*, which are AV/BV associated bacteria (Donders et al., 2017). Surprisingly, LEfSe results also revealed a higher abundance of *L. iners* and a lower abundance of *L. crispatus* in the higher grades group. This was consistent with our previous results that women with higher abundance of *L. iners* (CST III/I–III) had a higher proportion of vaginal inflammation, implying the greater risk of developing vaginal inflammation. Although a small sample size may be subject to some criticisms, the results still suggested the exclusive identity of *L. iners* (Petrova et al., 2017). Compared with other species of *Lactobacillus* (especially *L. crispatus*), *L. iners* possesses a poorer ability to produce the antibacterial product H_2_O_2_ and lactic acid (Kim et al., 2006; Petrova et al., 2017). Moreover, *L. iners* may even produce a toxin called Inerolysin similar to *Gardnerella vaginalis* (Rampersaud et al., 2018; Rampersaud et al., 2011). Therefore, in some cases, CST III has been described as a transitional state, sometimes associated with BV (Verstraelen et al., 2009; Walther-Antonio et al., 2014).

This study also explored the possible association of VMB and pregnancy outcomes. However, due to the limited sample size and a medium proportion of delivery data not collected, the results were uncertain. Since a lower BMI and the rate of passive smoking were also reportedly related to adverse pregnancy outcomes, these two may act as the modifications between VMB and adverse pregnancy outcomes (Wen et al., 2014). Moreover, CST III has been reported associated with preterm birth in some studies (Kindinger et al., 2017; Petricevic et al., 2014), further indicating the specialty of *L. iners* as we’ve mentioned previously. However, the proportion of CST III was high among Asian women, but the incidence rate of preterm birth was not higher than the western countries. We intend to expand our cohort in future research.

Our study had several limitations. Firstly, our study detected very little *G. vaginalis* and no BVAB1, which seemed different form other studies. However, due to the low proportion of *G. vaginalis* vagitypes among pregnant Asian women (3.6% in Korea by Kim et al. (2017), and 2/24 in Japan by Matsumoto et al. (2018)), we have to suppose that our participants in this study happened to have no vagitype of *G. vaginalis*. As for BVAB1, we did not really report it yet, probably due to the primer and reference database selection bias (Van Der Pol et al., 2019). Further researches may extend the reference database in advance. Moreover, our study only used a single sampling point, failing to explore the stability of the VMB throughout the pregnancy. Larger sample sizes and better study design are needed.

## Conclusions

The vaginal flora of asymptomatic pregnant Chinese women was mostly dominated by *L. crispatus* and *L. iners*. A lower BMI and more passive smoking may contribute to a less lactobacilli-dominant VMB. The investigation of the profile and influencers among pregnant women provides a basis for a further probe into the association of VMB and pregnancy outcomes. However, larger sample sizes are needed.

##  Supplemental Information

10.7717/peerj.8172/supp-1 Table S1 Grading of “vaginal cleanliness” by wet mount microscopy

10.7717/peerj.8172/supp-2 Table S2 Comparative analysis between women of delivery data collected and not collected *P*-values were calculated using chi-squared or Fisher’s exact analysis (*) for assessment of association of frequency between groups and the Mann–Whitney *U*-Test for comparison of means and medians . SD = standard deviation; BMI = Body Mass Index.

10.7717/peerj.8172/supp-3 Table S3 Relative abundance of top taxa in different CSTs CST = community state type; LD = lactobacilli-dominant group; LLD = less lactobacilli-dominant group.

10.7717/peerj.8172/supp-4 Table S4 Comparative analysis of relevant characteristics of subjects in different CSTs *P*-values were calculated using chi-squared or Fisher’s exact analysis (*) for assessment of association of frequency between groups and the Mann–Whitney *U*-Test for comparison of means and medians. CST = community state type; LD = lactobacilli-dominant group; LLD = less lactobacilli-dominant group; SD = standard deviation; BMI = Body Mass Index; IQR = interquartile range.

10.7717/peerj.8172/supp-5 File S1 Raw data—ASV table

10.7717/peerj.8172/supp-6 File S1 Raw data—Taxonomy assignments

## Additional Information and Declarations

### Data Availability

The following information was supplied regarding data availability: Data is available at NCBI Sequence Read Archive (SRA), accession number SRP216638.
