# Supplementary material for: Exploring profile and potential influencers of vaginal microbiome among asymptomatic pregnant Chinese women
Source: PeerJ. 2019 Dec 10;7:e8172. doi: 10.7717/peerj.8172 (PMC6910115; doi:10.7717/peerj.8172)
Supplement: Table S1 [file peerj-07-8172-s001.docx]

**Supplemental Table S1 Grading of “vaginal cleanliness” by wet mount microscopy**

| **Grades** | **Bacillus** | **Coccus** | **Epithelial cell** | **Leukocytes** |
| --- | --- | --- | --- | --- |
| I | ++++ | - | ++++ | 0-5/HP |
| II | ++ | - | ++ | 5-15/HP |
| III | - | ++ | - | 15-30/HP |
| IV | - | ++++ | - | >30/HP |
