# Supplementary material for: Exploring profile and potential influencers of vaginal microbiome among asymptomatic pregnant Chinese women
Source: PeerJ. 2019 Dec 10;7:e8172. doi: 10.7717/peerj.8172 (PMC6910115; doi:10.7717/peerj.8172)
Supplement: Table S2 — P-values were calculated using chi-squared or Fisher’s exact analysis (*) for assessment of association of frequency between groups and the Mann–Whitney U-Test for comparison of means and medians . SD = standard deviation; BMI = Body Mass Index. [file peerj-07-8172-s002.docx]

**Supplemental Table S2 Comparative analysis between women of delivery data collected and not collected**

| Characteristics (N=113) | Delivery data collected | Delivery data not collected | *P* |
| --- | --- | --- | --- |
|  | n=82 | n=31 |  |
| **Sociodemographic** |  |  |  |
| Age (mean ± SD) (years old) | 26.10 ± 3.91 | 24.61 ± 2.82 | 0.056 |
| Gestational age of weeks at enrollment (mean ± SD) (weeks) | 16.74 ± 2.75 | 16.52 ± 2.82 | 0.705 |
| Education status (Middle school or lower) | 19(23.2) | 10(32.3) | 0.324 |
| Economic status (<100,000 per year) | 38(46.3) | 12(38.7) | 0.466 |
| **Medical and reproductive history** |  |  |  |
| Maternal pre-pregnancy BMI (mean ± SD) (kg/m^2^) | 20.82 ± 2.69 | 21.22 ± 2.88 | 0.492 |
| Unipara | 53(64.6) | 24(77.4) | 0.193 |
| Presence of inflammation | 23(28.0) | 3(9.7) | 0.038 |
| Previous adverse pregnancy outcomes | 22(26.8) | 5(16.1) | 0.234 |
| **Lifestyle** |  |  |  |
| Vaginal douching | 23(28.0) | 14(45.2) | 0.084 |
| Active smoking | 4(4.9) | 4(12.9) | 0.212* |
| Passive smoking (>3 days per week) | 13(21.0) | 7(35.0) | 0.237* |
| Drinking | 6(7.3) | 3(9.7) | 0.704* |

*P*-values were calculated using chi-squared or Fisher’s exact analysis (*) for assessment of association of frequency between groups and the Mann–Whitney U-Test for comparison of means and medians. SD= standard deviation; BMI= Body Mass Index.
