# Supplementary material for: Exploring profile and potential influencers of vaginal microbiome among asymptomatic pregnant Chinese women
Source: PeerJ. 2019 Dec 10;7:e8172. doi: 10.7717/peerj.8172 (PMC6910115; doi:10.7717/peerj.8172)
Supplement: Table S3 — CST = community state type; LD = lactobacilli-dominant group; LLD = less lactobacilli-dominant group. [file peerj-07-8172-s003.docx]

**Supplemental Table S3 Relative abundance of top taxa in different CSTs**

| **Taxa** | **LD Group** | | | |  |  | **LLD Group** | |
| --- | --- | --- | --- | --- | --- | --- | --- | --- |
|  | **I** | **I-III** | **II** | **III** | **V** |  | **IV-A** | **IV-B** |
| *Lactobacillus crispatus* | 0.96572 | 0.52692 | 0.01619 | 0.02893 | 0.03439 |  | 0.05704 | 0.16834 |
| *Lactobacillus iners* | 0.01763 | 0.41918 | 0.13119 | 0.91950 | 0.32364 |  | 0.34444 | 0.10977 |
| *Lactobacillus gasseri* | 0.00261 | 0.00700 | 0.84511 | 0.01554 | 0.00253 |  | 0.00425 | 0.01297 |
| *Lactobacillus jensenii* | 0.00562 | 0.01615 | 0.00182 | 0.00813 | 0.62826 |  | 0.00485 | 0.01665 |
| *Megasphaera* | 0.00021 | 0.00033 | 0.00015 | 0.01362 | 0.00064 |  | 0.41949 | 0.00924 |
| *Dialister* | 0.00021 | 0.00789 | 0.00005 | 0.00375 | 0.00046 |  | 0.11225 | 0.16301 |
| *Lactobacillus reuteri* | 0.00207 | 0.01431 | 0.00149 | 0.00155 | 0.00508 |  | 0.00000 | 0.00000 |
| *Aerococcus* | 0.00002 | 0.00000 | 0.00268 | 0.00304 | 0.00005 |  | 0.03350 | 0.08671 |
| *Gardnerella* | 0.00049 | 0.00172 | 0.00000 | 0.00117 | 0.00106 |  | 0.00227 | 0.00000 |
| *Pseudomonas* | 0.00060 | 0.00025 | 0.00004 | 0.00065 | 0.00046 |  | 0.00392 | 0.00000 |
| *Gemella* | 0.00001 | 0.00000 | 0.00006 | 0.00009 | 0.00014 |  | 0.00010 | 0.12867 |
| *Enterococcus* | 0.00005 | 0.00000 | 0.00000 | 0.00017 | 0.00079 |  | 0.00000 | 0.07750 |
| *Ureaplasma* | 0.00024 | 0.00056 | 0.00025 | 0.00071 | 0.00000 |  | 0.00295 | 0.01634 |
| *Acinetobacter* | 0.00099 | 0.00004 | 0.00000 | 0.00011 | 0.00028 |  | 0.00000 | 0.01322 |
| *Stenotrophomonas* | 0.00024 | 0.00007 | 0.00000 | 0.00010 | 0.00022 |  | 0.00226 | 0.01186 |
| *Streptococcus* | 0.00013 | 0.00000 | 0.00007 | 0.00032 | 0.00004 |  | 0.00000 | 0.01758 |
| *Prevotella* | 0.00010 | 0.00046 | 0.00000 | 0.00029 | 0.00029 |  | 0.00383 | 0.00000 |
| *Lactobacillus buchneri* | 0.00003 | 0.00000 | 0.00000 | 0.00003 | 0.00014 |  | 0.00000 | 0.03960 |
| *Mycoplasma* | 0.00000 | 0.00355 | 0.00000 | 0.00026 | 0.00000 |  | 0.00020 | 0.00000 |
| *Lactococcus* | 0.00033 | 0.00057 | 0.00000 | 0.00023 | 0.00032 |  | 0.00062 | 0.00334 |

CST= community state type; LD= lactobacilli-dominant group; LLD= less lactobacilli-dominant group.
