# Supplementary material for: Exploring profile and potential influencers of vaginal microbiome among asymptomatic pregnant Chinese women
Source: PeerJ. 2019 Dec 10;7:e8172. doi: 10.7717/peerj.8172 (PMC6910115; doi:10.7717/peerj.8172)
Supplement: Table S4 — P-values were calculated using chi-squared or Fisher’s exact analysis (*) for assessment of association of frequency between groups and the Mann–Whitney U-Test for comparison of means and medians. CST = community state type; LD = lactobacilli-dominant group; LLD = less lactobacilli-dominant group; SD = standard deviation; BMI = Body Mass Index; IQR = interquartile range. [file peerj-07-8172-s004.docx]

**Supplemental Table S4 Comparative analysis of relevant characteristics of subjects in different CSTs**

| Characteristics (N=113) | LD Group (n=100) | |  | LLD Group (n=13) | *P* |
| --- | --- | --- | --- | --- | --- |
|  | CST I/II/V (n=58) | CST I-III/III (n=42) |  | CST IV-A/B |  |
| **Sociodemographic** |  |  |  |  |  |
| Age (mean ± SD) (years old) | 25.88 ± 3.81 | 25.45 ± 3.77 |  | 25.62 ± 3.07 | 0.850 |
| <18 | 1(1.7) | 0(0.0) |  | 0(0.0) | 0.890* |
| 18-25 | 19(32.8) | 17(40.5) |  | 5(38.5) |  |
| 25-35 | 38(65.5) | 25(59.5) |  | 8(61.5) |  |
| Gestational age of weeks at enrollment (mean ± SD) (weeks) | 16.90 ± 3.13 | 16.61 ± 2.47 |  | 15.92 ± 1.64 | 0.506 |
| 12-18 | 46(79.3) | 33(78.6) |  | 12(92.3) | 0.841* |
| 18-24 | 9(15.5) | 8(19.0) |  | 1(7.7) |  |
| 24-28 | 3(5.2) | 1(2.4) |  | 0(0.0) |  |
| Education status |  |  |  |  | 0.825* |
| Middle school and lower | 14(24.1) | 11(26.2) |  | 4(30.8) |  |
| High school and higher | 44(75.9) | 31(73.8) |  | 9(69.2) |  |
| Economic status (RMB per year) |  |  |  |  | 0.760 |
| <100,000 | 25(43.1) | 18(42.9) |  | 7(53.8) |  |
| >=100,000 | 33(56.9) | 24(57.1) |  | 6(46.2) |  |
| **Medical and reproductive history** |  |  |  |  |  |
| Maternal pre-pregnancy BMI (mean ± SD) (kg/m^2^) | 21.37 ± 2.69 | 20.88 ± 2.67 |  | 19.16 ± 2.60 | 0.030 |
| Underweight (<18.5) | 6(10.3) | 7(16.7) |  | 5(38.5) | 0.209* |
| Normal weight (18.5-24.0) | 42(72.4) | 29(69.0) |  | 7(53.8) |  |
| Overweight and obese (>24.0) | 10(17.2) | 6(14.3) |  | 1(7.7) |  |
| Unipara | 37(63.8) | 32(76.2) |  | 8(61.5) | 0.343* |
| No | 21(36.2) | 10(23.8) |  | 5(38.5) |  |
| Presence of inflammation (Grade III/IV) | 7(12.1) | 15(35.7) |  | 4(30.8) | 0.012* |
| No | 51(87.9) | 27(64.3) |  | 9(69.2) |  |
| Previous adverse pregnancy outcomes | 17(29.3) | 7(16.7) |  | 3(23.1) | 0.310* |
| No | 41(70.7) | 35(83.3) |  | 10(76.9) |  |
| **Lifestyle** |  |  |  |  |  |
| Vaginal douching | 26(44.8) | 15(35.7) |  | 3(23.1) | 0.648 |
| Never | 32(55.2) | 27(64.3) |  | 10(76.9) |  |
| Active smoking | 3(5.2) | 3(7.1) |  | 2(15.4) | 0.369* |
| Never | 55(94.8) | 39(92.9) |  | 11(84.6) |  |
| Passive smoking (>3 days per week) | 7(12.1) | 8(19.0) |  | 5(38.5) | 0.086* |
| <=3 days per week | 51(87.9) | 34(81.0) |  | 8(61.5) |  |
| Drinking | 5(8.6) | 3(7.1) |  | 1(7.7) | >0.99* |
| Never | 53(91.4) | 39(92.9) |  | 12(92.3) |  |
| **Alpha diversity** |  |  |  |  |  |
| Shannon index (median [IQR]) | 0.23 [0.15, 0.37] | 0.44 [0.24, 0.87] |  | 2.30 [1.69, 2.48] | <0.001 |

*P*-values were calculated using chi-squared or Fisher’s exact analysis (*) for assessment of association of frequency between groups and the Mann–Whitney U-Test for comparison of means and medians. CST= community state type; LD= lactobacilli-dominant group; LLD= less lactobacilli-dominant group; SD= standard deviation; BMI= Body Mass Index; IQR= interquartile range.
